# Generation of Synthetic Chest X-ray Images and Detection of COVID-19: A Deep Learning Based Approach

**DOI:** 10.3390/diagnostics11050895

**Published:** 2021-05-18

**Authors:** Yash Karbhari, Arpan Basu, Zong Woo Geem, Gi-Tae Han, Ram Sarkar

**Affiliations:** 1 Department of Information Technology, Pune Vidyarthi Griha’s College of Engineering and Technology, Pune 411009, India; yashkarbhari17@gmail.com; 2 Department of Computer Science and Engineering, Jadavpur University, Kolkata 700032, India; arpan0123@gmail.com (A.B.); ramjucse@gmail.com (R.S.); 3 College of IT Convergence, Gachon University, 1342 Seongnam Daero, Seongnam 13120, Korea; gthan@gachon.ac.kr

**Keywords:** COVID-19 detection, generative adversarial network, synthetic data generation, harmony search, feature selection, chest X-ray, deep learning

## Abstract

COVID-19 is a disease caused by the SARS-CoV-2 virus. The COVID-19 virus spreads when a person comes into contact with an affected individual. This is mainly through drops of saliva or nasal discharge. Most of the affected people have mild symptoms while some people develop acute respiratory distress syndrome (ARDS), which damages organs like the lungs and heart. Chest X-rays (CXRs) have been widely used to identify abnormalities that help in detecting the COVID-19 virus. They have also been used as an initial screening procedure for individuals highly suspected of being infected. However, the availability of radiographic CXRs is still scarce. This can limit the performance of deep learning (DL) based approaches for COVID-19 detection. To overcome these limitations, in this work, we developed an Auxiliary Classifier Generative Adversarial Network (ACGAN), to generate CXRs. Each generated X-ray belongs to one of the two classes COVID-19 positive or normal. To ensure the goodness of the synthetic images, we performed some experimentation on the obtained images using the latest Convolutional Neural Networks (CNNs) to detect COVID-19 in the CXRs. We fine-tuned the models and achieved more than 98% accuracy. After that, we also performed feature selection using the Harmony Search (HS) algorithm, which reduces the number of features while retaining classification accuracy. We further release a GAN-generated dataset consisting of 500 COVID-19 radiographic images.

## 1. Introduction

The new virus, SARS-CoV-2, has resulted in a worldwide pandemic where the affected individuals suffer from respiratory diseases. This has resulted in large-scale social and economic disruptions. Coronavirus typically leads to an upper respiratory infection (URI). Mild symptoms include fever, dry cough, breathing difficulties, etc. Complications may result in pneumonia and ARDS. More than 80 million cases have been reported to date and more than 1.8 million deaths have occurred due to COVID-19. According to Nicholls et al. [1], on 8 December 2020, a new variant with 23 new mutations was discovered. This finding has raised concerns worldwide because vaccines were being launched for emergencies during that time. A study by Chen et al. [2] shows that the radiographic images of infected individuals have significant artifacts that can be used for diagnosis. CXR is one of the important non-invasive clinical techniques that can be used to detect such artifacts associated with COVID-19 disease. It is to be noted that CXR is sensitive, but not specific. The same abnormalities in the CXR may be produced by some other lung infections. At the same time, CXR is more likely to be useful in staging lung disease severity [3,4,5,6,7]. Hence it is one of the important tools in the treatment of COVID-19. Studies by Nicholls et al. [1] also show that the CXR does not have a major impact with a difference in the type of mutation. Many radiologists have been using CXR images as a key component to detect COVID-19. However, some hindrances are faced while developing computer-aided diagnosis systems to detect COVID-19 in CXR images. The most important factors are the scarcity of expert radiologists to decipher the CXR images and the minute details in the radiographic responses of patients. The idea of detecting COVID-19 by a machine quickly comes to mind and might seem effective and feasible as it will lower the costs due to less labor being needed. The main issue to train a machine learning based algorithm, in this case, is the availability of a large quantity of data. Many DL based CNN models have been used in classifying COVID-19 CXR images and also in the field of medical imaging. However, there are still many challenges for CNNs to be completely reliable. One of the major challenges is the availability of data. Studies have shown that a large quantity of data is essential for the better generalizability and performance of CNNs.

To solve this problem of data unavailability, many deep learning (DL) models have been created, and the results seem to detect COVID-19 correctly using CXR images. For example, the authors of the works reported in [8,9,10] have proposed methods for detecting COVID-19 from radiological images. In addition to the previous works, there are also works that have used CNNs for COVID-19 classification in the multi-class setting. In most cases, the COVID affected and normal CXRs are combined with pneumonia affected CXRs from another dataset, and this combined dataset is then used for training the models. Examples include the works by Apostolopoulos and Mpesiana [11] and Kikkisetti et al. [12]. Another recent work by Ozturk et al. [13] used a DarkNet based classifier and achieved accuracies of 98.08% and 87.02% in the binary and multi-class settings, respectively. A similar work by Saha et al. [14] used a graph isomorphic network based model and achieved accuracy scores of above 99% in the multi-class setting. A work by Das et al. [15] also tackled the multi-class classification task by using a bi-level model on features extracted from a CNN.

In this paper, we attempt to solve the above problem of data unavailability in COVID-19 CXRs by generating synthetic images using data augmentation. Data augmentation is a process that is used to create new data samples. The existing data are also used so that the artificially generated data remains similar to the original samples. This is done by applying techniques such as random rotation, random brightness, etc. to samples from the training set to create synthetic samples that are similar to the original samples. However, simple data augmentation cannot be used to generate completely unseen data, as the augmentation is based on the existing training data. The output from such simple augmentation methods will be very similar to the input data provided. Therefore, in the present work, we rely on a Generative Adversarial Network (GAN) to produce completely new training samples.

The use of such data augmentation techniques to produce synthetic samples for COVID-19 has already been reported in the literature. Waheed et al. [16] developed an ACGAN based approach for generating synthetic CXRs of COVID-19 affected and normal individuals. Such a stage improves the performance in a downstream classifier when the synthetic images are used in addition to the origin images. A similar approach was used by Loey et al. [17], where the authors utilized a conditional GAN for data augmentation. They have shown the performance improvement by considering some state-of-the-art CNNs. Rajaraman and Antani [18] proposed another approach where the training dataset is expanded using weakly labelled CXR images from publicly available pneumonia datasets. Initially, some CNNs are trained to predict bacterial and viral pneumonia on a baseline dataset. Thereafter, the best model from the above training process is used to weakly label CXRs in other datasets. Finally, the baseline is augmented with the weakly labelled CXRs, and a performance improvement is observed in this case.

GANs were first introduced by Goodfellow et al. [19] and have been in use for a while now. They have been used to generate more unseen data in the field of medical imaging. Many variants of GAN have been developed to stabilize the GAN and to increase its performance with minimum computational power. GANs have attained state-of-the-art performance in many domains related to image processing. In the present work, we use an Auxiliary Classifier Generative Adversarial Network (ACGAN) to generate synthetic images of CXRs. In addition to the noise, an ACGAN also takes the class label (COVID-19 positive CXR or normal CXR) as the input to generate the corresponding image belonging to the specified class. Taking reference from recent works [20], label smoothing is used to discourage the model from overfitting. We use a dataset consisting of 584 COVID CXRs and 1232 normal CXRs collected from multiple sources for training the GAN.

After synthetic data generation, the produced CXR images are used to train several state-of-the-art CNNs for COVID-19 detection. This has been done to judge the quality of the generated images as compared to the original data. In this work, we have considered the VGG16 [21], VGG19, ResNet50 [22], Xception [23] and InceptionV3 [24] models for this purpose. In addition, feature selection was also performed using a popular meta-heuristic algorithm known as Harmony Search (HS) [25], on the CNN extracted features. This increases the classification accuracy while decreasing the number of features. The CNN features may contain some redundant features, which decreases the classification accuracy. This is mitigated by such a feature selection stage.

The major points of this work are as follows:

1. An ACGAN based approach is applied to generate synthetic CXRs of normal and COVID-19 affected patients.
2. Label smoothing is applied in the ACGAN.
3. Some recent CNN based classifiers are trained on the synthetic images for detecting COVID-19 in the CXR.
4. Feature selection is performed on the CNN extracted feature vector using the HS algorithm for reducing the dimension of the feature vector.

The remainder of the paper is organized into the following sections: Section 2 details some prior work related to the topic. Section 3 discusses the present approach. Section 4 and Section 5 presents the results and some related discussion, respectively. Finally, we end with concluding our work in Section 6.

## 2. Related Work

The last decades have witnessed the adoption of DL in various domains, including medical image processing. This is mainly due to the significant growth in computing power and also due to the availability of large-scale datasets. Many works have been proposed in domains such as image classification, video summarization, image captioning, image retrieval [26], computer-aided diagnosis, etc., which utilize DL based methods such as CNNs, GANs, etc.

GANs were first proposed in the work by Goodfellow et al. [19]. GANs consist of two models that are trained together. The first model is called the generator *G* that learns the input data distribution. The second model is called the discriminator *D*. It predicts the probability that an input image is produced by *G* or is an original sample from the training dataset. A GAN can be viewed as a two-player minimax game, wherein *G* maximizes the probability of *D* making a mistake and *D* minimizes the same probability.

Since the initial paper regarding GAN, various modifications and improvements have been proposed in the literature. Of particular interest is the ACGAN [27] model, which introduces class conditioning in the vanilla GAN. The authors have highlighted in their paper that this leads to better global coherence and hence better performance as compared to the vanilla GAN. An added benefit is that we can fix the output class while generating the output images.

Several authors have also successfully applied GANs to achieve good results in various tasks such as activity recognition [28], image separation [29], etc. Specifically, GANs have also been used widely in the field of medical image processing. For instance, Armanious et al. [30] proposed MedGAN, which can be applied to medical image processing tasks such as PET/CT translation, image denoising, etc. The adversarial loss has been combined with non-adversarial and style-transfer loss. Additionally, a novel generator architecture consisting of multiple encoder–decoder networks has been utilized by the authors for progressive refinement of the inputs. Similarly, Woternik et al. [31] used the Pix2Pix framework for denoising low-dose CT scans. The authors noted that training the CNNs with both voxelwise loss and adversarial loss made it possible to capture the image statistics of routine-dose images better.

The lack of data in the domain of medical imaging is still a substantial problem being addressed by researchers. Han et al. [32] proposed a novel idea for data augmentation with GANs to generate brain Magnetic Resonance Imaging (MRI) for tumor detection. The authors noted the advantages of noise-to-image and image-to-image GANs, and have combined the two approaches to obtain synthetic images of better quality. The approach consists of two stages. In the first stage, Progressive Training for GANs (PGGANs) is applied to generate realistic and diverse synthetic MRIs. The second stage consists of Multimodal UNsupervised Image-to-image Translation (MUNIT) that improves the quality of the images generated in the first stage such that they resemble real MRIs. MUNIT utilizes GANs or variational autoencoders in order improve the texture of the images generated by the PCGAN. The inclusion of the results in a downstream ResNet50 classifier improves the performance of the model. The authors have observed a marked improvement in the accuracy, sensitivity and specificity of the classification results.

The work by Dirvanauskas et al. [33] is another one in the domain of medical image synthesis. The authors developed a human embryo image generator based on GANs for generating synthetic images of human embryo cells. These data can later be used in downstream tasks (training models, classification, etc.) for embryo image processing. The authors generated one-cell, two-cell and four-cell embryo images, and achieved a misclassification rate of 12.3% on the synthetic images. Furthermore, expert evaluation indicated the true recognition rates as 96.2%, 86.8% and 80.00% for the three types of images, respectively. The authors have also shown that there were no statistically significant differences between the real and synthetic images, which highlights the effectiveness of their method.

The lack of data availability of CXR images for COVID-19 and the success of GANs for data synthesis have provided a new direction for research. In line with this statement, Waheed et al. [16] proposed an approach based on ACGAN, which they term as CovidGAN. The generator in CovidGAN takes a noise vector and the class label (COVID-19 positive or negative) as input, and outputs an image of a CXR. The image then progresses to the discriminator and produces two outputs: whether the image is real or fake and whether it is a COVID-CXR or NORMAL-CXR. The generator consists of 22 million parameters and the discriminator consists of 2 million parameters. The authors have used a mixture of three datasets: the IEEE Covid CXR dataset, the COVID-19 Radiography database, and the COVID-19 CXR dataset Initiative. The compiled dataset contains 1124 CXRs, of which 403 are COVID-19 positive and 721 are normal. The authors used the synthetic images in a downstream VGG16 classifier, which improved the accuracy from 85% to 95%.

Here, we would like to note that GANs have also been applied for tasks other than image synthesis from input noise vectors. Image-to-image translation [34] is an important application of GANs. This has been applied to many tasks, such as super-resolution [35], binarization [36,37], document analysis [38,39], etc. In addition to the above, many CNN based approaches have also been proposed for COVID-19 detection in radiological images. We highlight some of them below. As noted previously, their performance is highly dependent on the size of the datasets.

Jaiswal et al. [9], in their work, applied the concept of transfer learning in their DL models. The authors first trained the models on the ImageNet dataset. Thereafter, the pretrained models were trained on the SARS-CoV-2 CT-scan dataset to classify the input images into the two classes of COVID-19 infected and negative. The authors observed that the DenseNet architecture provides better results compared to other architectures such as VGG, ResNet, and InceptionResNet. An accuracy score of 96.25% was reported on the test set.

Goel et al. [40] introduced an optimized CNN (OptCoNet) for diagnosis of COVID-19. The basic architecture of the model is that of a conventional CNN comprised of convolutional, pooling, dense, and fully-connected layers. However, the authors used the Grey Wolf Optimization (GWO) algorithm to tune the CNN. The authors showed that their approach performs better when compared to some state-of-the-art CNNs. The reported accuracy and F1 score values were 97.78% and 95.25%, respectively.

Rajaraman et al. [41], in their work, demonstrated the use of pruning and ensembling to enhance the performance of their DL models. The authors developed a framework for detecting COVID-19-related irregularities in CXRs. They have also applied modality-specific training on pneumonia-related data in addition to pretraining on ImageNet to improve the classification accuracy. Iterative pruning was then performed on the best models. This helped decrease the complexity of the models, and improved the memory efficiency at the same time. The authors reported the accuracy as 99.01% and the AUC score as 0.9972.

Gianchandani et al. [42] also used a similar ensemble based approach. They utilized two datasets for training the DL models. The first one was obtained from Kaggle and used for binary classification. It contained three classes, namely COVID positive, COVID negative, and pneumonia, from which the first two classes were selected. The second dataset was used for multiclass classification. It was collected by a research team in collaboration with doctors. The authors reported the accuracies as 96.15% and 99.21% for the binary and multi-class classification tasks, respectively.

Murugan and Goel [43] proposed an extreme machine learning based deep learning classifier model (E-DiCoNet) for COVID-19 diagnosis. The proposed algorithm, being non-iterative in nature, takes less time than the iterative backpropagation based approaches. They used a dataset consisting of 2700 CXRs collected from various public repositories. There were three classes, namely COVID CXR, normal CXR and pneumonia affected CXR, with 900 images in each class. The authors reported the accuracy and F1 score values as 94.07% and 91.22%, respectively.

Apart from the above CNN based classifiers, the work by Garain et al. [44] also explored the use of spiking neural networks (SNNs) to detect COVID-19 in chest computed tomography (CT) scan images. The main impetus behind using SNNs is the development of neuromorphic chips in recent times. Such chips will offer better performance and efficiency for SNNs as compared to DL algorithms on conventional Graphics Processing Units (GPUs). The main disadvantage compared to GPU-trained DL algorithms would be the huge training time of SNN based approaches. The work explored the use of two kinds of SNNs, potential based and spike based, and found that the former performed much better than the latter. Furthermore, it also achieved an F1 score of 0.99, outperforming several state-of-the-art CNN based models such as VGG, ResNet and DenseNet.

Feature selection is the process of choosing a subset of relevant features for improving model performance. The features produced by CNNs generally have redundancies among them, which may reduce the final prediction accuracy. In many cases (as in [45]), using a machine learning classifier after performing feature selection can improve the overall performance of the learning system. The HS algorithm [25] is a meta-heuristic algorithm that has been widely used as a feature selection method in various research works. In a work by Saha et al. [46], cosine similarity was used along with HS for facial emotion recognition. Similarly, in a work by Sarkar et al. [47], HS was used for microstructural image classification. Several works [48,49] also exist that have hybridized HS along with other optimization algorithms. This highlights the utility of HS as a competent feature selection algorithm.

In the above discussion, we have considered several works related to the use of GANs for medical image generation, CNN based approaches for COVID-19 detection, and application of feature selection in different domains. In particular, GANs are a useful tool to generate high-quality synthetic data irrespective of the domains, and researchers across the world have been utilizing this where there is a lack of required data. These synthetic samples are better than conventional samples produced by vanilla data augmentation techniques, such as rotation, cropping, brightness modification, etc. However, there are some challenges. The input data must be varied enough, otherwise the generated images will be similar in nature with almost identical texture, and lack variety. The training of GANs can also be unstable at times, which can lead to mode collapse. Despite their limitations, GANs are a useful tool for medical image generation, and active research is ongoing to improve the quality of images as well as the stability of GANs. Hence, we considered the use of GAN in the present work to generate CXRs. Obtained high-quality images can be helpful in domains with very small amounts of data, and especially for COVID-19 detection.

## 3. Materials and Methods

**Figure 1:**
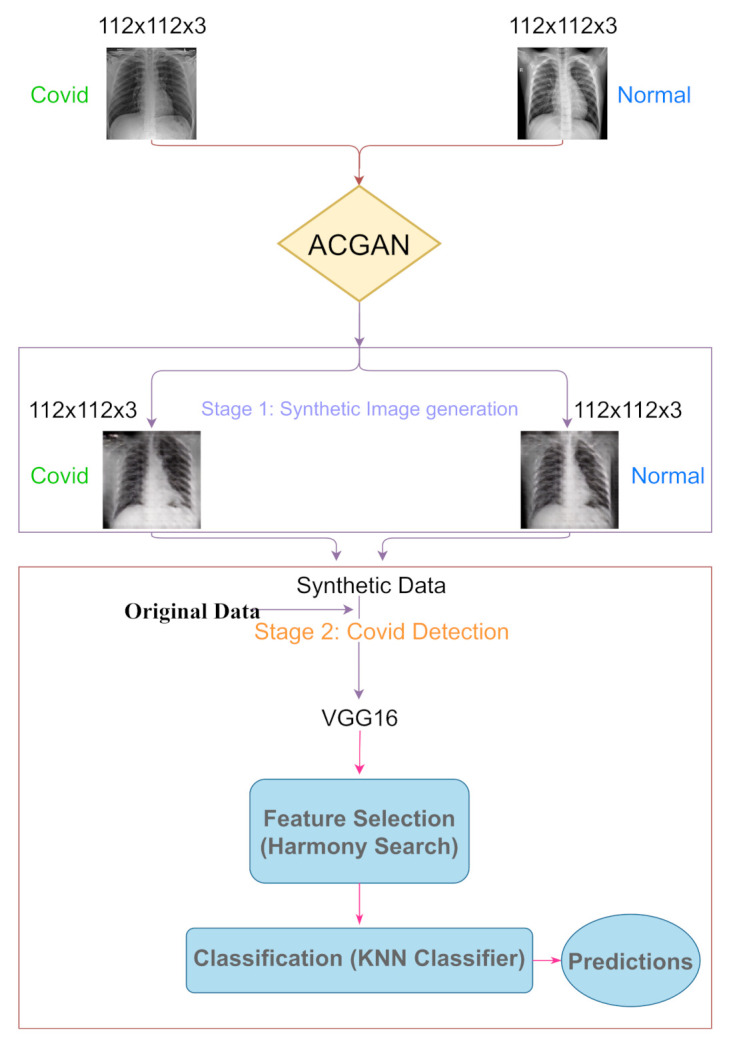
Flowchart representing the overall process.

Here we discuss the present approach. Figure 1 provides a pictorial outline of the entire process. In the following sections, we elaborate more on the individual stages.

### 3.1. Synthetic Image Generation

**Figure 2:**
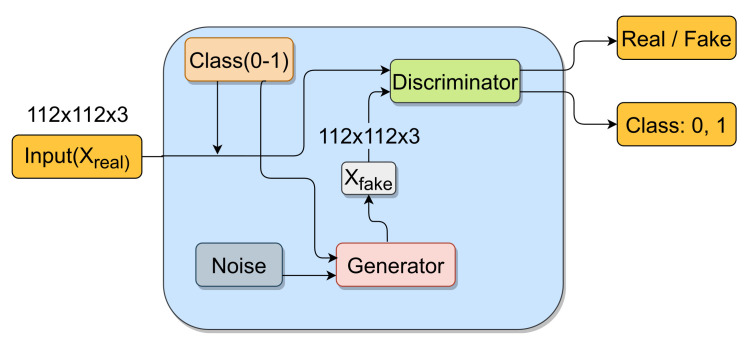
Schematic diagram of ACGAN.

In this section, we describe the overall process that is used for synthetic image generation. The basic architecture is the same as that of an ACGAN [27]. Figure 2 shows a schematic diagram of the GAN architecture. We also briefly summarize the generator and discriminator structures below.

#### 3.1.1. Generator

The generator takes a 50-dimensional noise vector *z* and an integer class label *c* as input. The noise is randomly sampled from a normal distribution having a mean of 0 and a standard deviation of 0.02. The class label is first passed through an embedding layer of 50 dimensions and then through a dense layer with 7×7 units to produce a 7×7×1 tensor. The noise vector is passed through a dense layer having 1024×7×7 units to produce a 7×7×1024 tensor. The two output tensors are then concatenated to produce a 7×7×1025 tensor. This is followed by four successive transposed convolutions to produce tensors with dimensions 14×14×512, 28×28×256, 56×56×128 and 128×128×3, respectively. Each transposed convolution is followed by batch normalization and Rectified Linear Unit (ReLU) activation, except in the last one, where tanh activation is used. The kernel size and the stride are 5 and 2, respectively, in the transposed convolution layers. The final output from the generator is an image $X_{fake}$ of dimension 112×112×3.

#### 3.1.2. Discriminator

The discriminator takes an image of shape 112×112×3 as input. The image is either from the original dataset ($X_{real}$) or is generated by the generator ($X_{fake}$). The discriminator is composed of four convolutional blocks. Each block consists of a convolutional layer, batch normalization layer, LeakyReLU activation (slope = 0.2) and a dropout layer (probability = 0.5) in succession. The blocks downsample the input image from a dimension of 112×112×3 to dimensions 56×56×64, 28×28×128, 14×14×256 and 7×7×512, respectively. Finally, the tensor is flattened and passed to two dense layers that produce the final two outputs.

The first dense layer produces a 1D tensor and has sigmoid activation associated with it. It is essentially a binary classifier producing a probability indicating whether the input image is from the original dataset (“real”) or it is produced by the generator (“fake”).

The second dense layer produces a 2D tensor (essentially a matrix) and has softmax activation associated with it. It is essentially a classifier identifying the class of each input image.

#### 3.1.3. Training

The Adam [50] optimizer is used for training both the generator and the discriminator. The learning rate is kept as 0.0002 and the beta value is kept as 0.5. Training is performed for 1200 iterations with a batch size of 36.

Let $X_{real}$ denote a “real” image from the dataset, and $X_{fake}=G\left(z,c\right)$ denote a “fake” image produced by the generator *G* on input noise *z* and class label *c*. The discriminator produces a probability distribution over the sources (real/fake) as well as the class label (COVID-CXR/Normal-CXR) given by P(S|X),P(C|X)=D(X). Here *S* and *C* denote the source and class predictions of the discriminator while *X* denotes the image that is passed to the discriminator. An ACGAN utilizes the following two parts in its objective function:

1. Source loss: The log likelihood of the correct source (see Equation (1)).
2. Class loss: The log likelihood of the correct class (see Equation (2)).

(1) $$L_{s}=E\left[logP\left(S=real|X_{real}\right)\right]+E\left[logP\left(S=fake|X_{fake}\right)\right]$$

(2) $$L_{c}=E\left[logP\left(C=c|X_{real}\right)\right]+E\left[logP\left(C=c|X_{fake}\right)\right]$$

The generator is trained to maximize $L_{c}-L_{s}$, whereas the discriminator is trained to maximize $L_{c}+L_{s}$.

#### 3.1.4. Label Smoothing

Label smoothing is applied in the GAN as shown in Equation (3). *L* and $L_{s}$ are the initial and smoothed labels, respectively. Rand represents the pseudo-random number generator function, which produces a number between 0 and 1. MaxDev is the scaling factor that scales the random number to have a value between 0 and MaxDev instead of having 1 as the upper bound. We use a MaxDev value of 0.2 in our experiments. The value of 0.2 for MaxDev was found experimentally. There are also works that have found success using similar values [51].

(3) $$L_{s}=\left\{\begin{array}{c} L+MaxDev\times Rand(),\;if\;L=0\\ L-MaxDev\times Rand(),\;if\;L=1 \end{array}\right.$$

Label smoothing converts the “hard” label assignments into “soft” labels. It is a regularization method that improves the generalizability of models and thus improves their performance, as noted in the work by Müller et al. [20].

### 3.2. COVID-19 Detection

In this section, we briefly summarize the different CNN architectures that are used to measure the goodness of the synthetic images as training samples generated by the ACGAN. Initially, the models were trained on the ImageNet dataset in a pretraining stage. Thereafter, models were trained for 20 epochs applying the Adam [50] optimizer. A learning rate value of 0.001 was used. We used categorical cross-entropy as the loss function and ReLU as the activation function for the intermediate layers. The batch size was kept as 16. We used 252 COVID-CXR and 432 NORMAL-CXR for the training set and 28 COVID-CXR and 48 NORMAL-CXR for the test set. The train–test split ratio was 90%:10%. The Keras library was used for implementation.

#### 3.2.1. VGG

The VGG [21] architecture is one of the relatively old and simple architectures. From a network perspective, it only uses the basic CNN layers. The last fully-connected layer has softmax activation and produces a one-dimensional tensor with the size being equal to the number of output classes.

#### 3.2.2. ResNet

**Figure 3:**
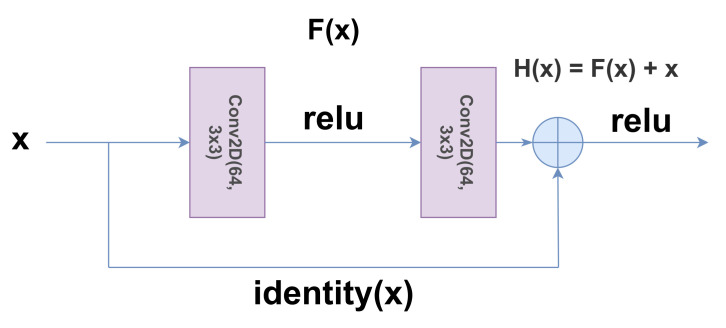
A schematic diagram of the skip connections in the ResNet architecture. (He et al. [22]).

The ResNet [22] architecture was first proposed to tackle the challenges that are faced while training very deep networks. Normally, with a very deep network, the problem of the vanishing gradients is experienced, where the magnitude of the gradients reduces to a very low value. ResNet consists of skip connections in between layers (see Figure 3), which diminishes this problem due to the identity connection thus produced. This greatly improves the performance of deep networks.

#### 3.2.3. Inception

**Figure 4:**
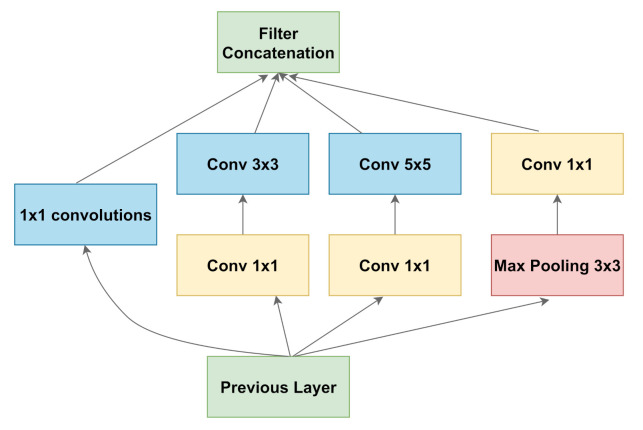
A schematic diagram representing an inception block. (Chollet [23]).

The Inception architecture is a comparatively complex architecture. The InceptionV3 architecture was proposed in Szegedy et al. [24], and it builds on the previous versions of the Inception architectures. The main intuition was that with a rethinking of the inception blocks, an efficient and complex network can be created with very few parameters as compared to the VGG architectures. The paper uses various techniques such as factorizing convolution, auxiliary classifier, label smoothing, etc. to achieve better performance and parameter efficiency. A schematic of the inception block is highlighted in Figure 4.

#### 3.2.4. Xception

**Figure 5:**
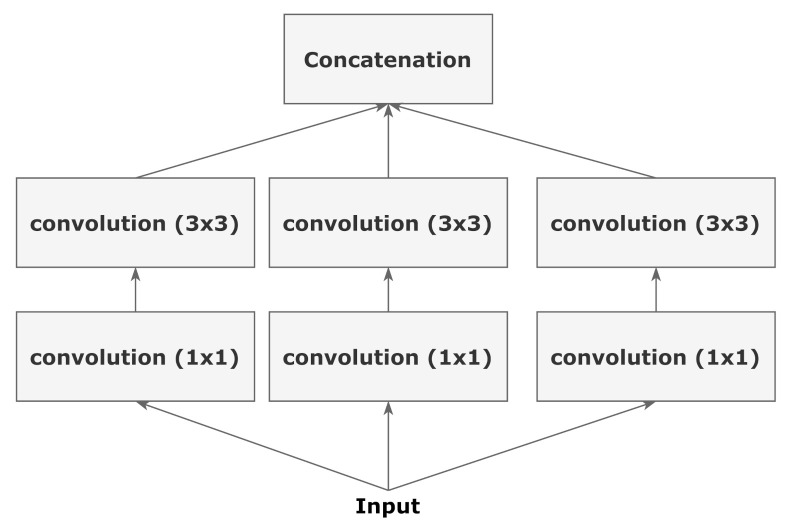
A schematic diagram representing a simplified inception block. Compared to Figure 4, it only contains a single size of convolutions (3×3) and does not contain pooling layers. (Chollet [23]).

**Figure 6:**
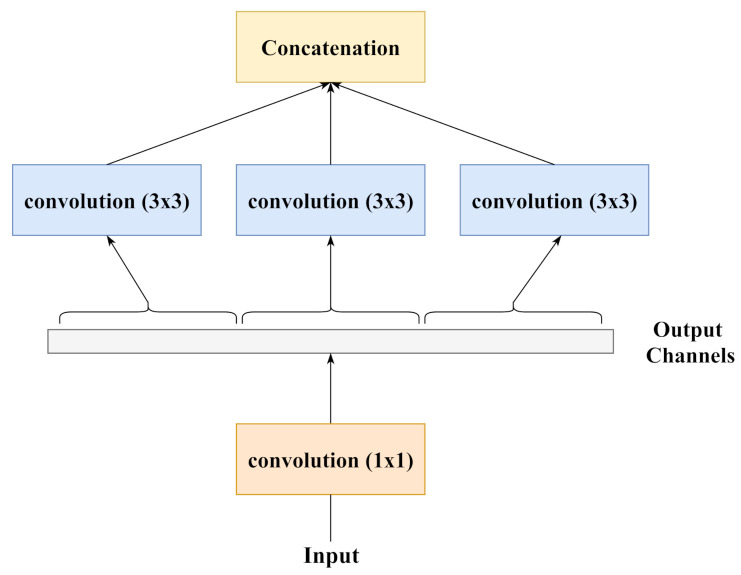
A strictly equivalent reformulation of the simplified inception block of Figure 5. Chollet [23].

The Xception [23] architecture presents an alternative view of inception modules as depthwise separable convolutions. Figure 5 and Figure 6 show a simple inception block and its equivalent reformulation, respectively. When the spatial convolutions in the reformulated block are applied to each individual output channel, the operation becomes equivalent to using depthwise separable convolutions. Those authors have noted that the Xception architecture performs better than the InceptionV3 architecture on the ImageNet dataset despite having approximately the same number of parameters. Hence, Xception makes more efficient use of the model parameters compared to InceptionV3.

### 3.3. Feature Selection

In this section, we examine the application of feature selection to reduce the dimensionality of the feature vectors obtained from the deep learning models with no adverse effect on the overall prediction capability of the models. First, the feature vectors are obtained by taking the output from the second-last dense layer of the DL model. This is the feature representation before the final output, which is the probability distribution of the classes. In our case, it is a one-dimensional tensor with a size of 128. These features are then fed to the feature selection stage.

**Figure 7:**
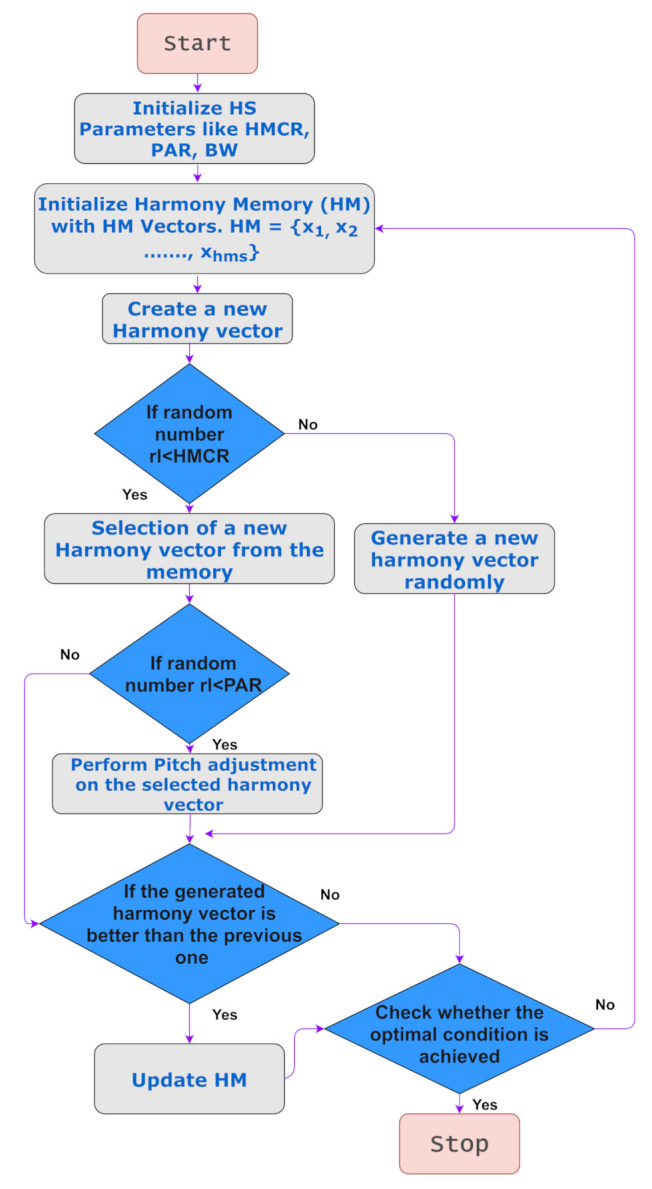
A flowchart representing the HS algorithm.

The HS algorithm proposed by Geem et al. [25] is utilized for feature selection. It was developed keeping musical harmony in consideration. Musical harmony comprises sounds that are considered pleasing from an aesthetic point of view. Musical performances seek to achieve the best harmony, and HS seeks to achieve the best state or global optimum. HS introduces a memory of sorts called the Harmony Memory (HM), which consists of the harmonies in sorted order of the objective function values. Figure 7 provides an outline of the HS algorithm.

The HS algorithm consists of the following steps:

1. Initialize an HM.
2. Devise a new harmony from the HM.
3. If the new harmony has a better fitness than the worst harmony, then replace the worst with the current harmony.
4. If the stopping criterion is met, then stop; else, continue from step 2.

The HS algorithm also has other parameters such as the Harmony Memory Consideration Rate (HMCR) and Pitch Adjusting Rate (PAR). HMCR is the probability of choosing a variable value from HM in the next step. PAR denotes the probability of the algorithm choosing a neighboring value. In the traditional HS algorithm, these parameters remain fixed, and this affects the performance of the algorithm. The work by Mahdavi et al. [52] aims to address these issues by making some of the parameters dynamic. In particular, the values are changed dynamically with the generation number, which results in some improvement over the basic HS algorithm.

The fitness function *F* that is used to determine the quality of a particular solution is highlighted in Equation (4). Here, *H* is the harmony for which the fitness is being calculated, λ is a weightage factor in the range [0,1], *a* is a function that computes the classification accuracy given a harmony *H*, |H| represents the number of selected features in the harmony *H*, and *T* denotes the total number of features. The fitness is to be maximized and is composed of a summation of two components. The left-hand part represents the accuracy component, and the right-hand part represents the feature selection component.

(4) $$F\left(H\right)=\lambda \;a\left(H\right)+\left(1-\lambda \right)\left(1-\frac{\left|H\right|}{T}\right)$$

In the present work, we have used the Py_FS (https://github.com/Ritam-Guha/Py_FS, accessed on 27 March 2021) package for the HS algorithm. The selected features were then used in a KNN classifier with five neighbors to obtain the final classification results. The value of λ was kept as 0.9 while the remaining parameters were used with the default values. The choice of the HS algorithm as because of its good trade-off between the exploitation and exploration phases. In addition, it has produced good results in our case. We evaluated the performance of HS with some other meta-heuristic based feature selection algorithms and found that HS provided the best results among these. The quantitative results are highlighted in Section 4. The HS algorithm follows some intuitive steps that are similar to how a musician composes a harmony. This, combined with the fact that it is easy to implement, makes it a good choice to find solutions to optimization problems. Thus, it has also been used in some recent works for feature selection [46,47,53,54].

### 3.4. Dataset Used

We assembled a dataset consisting of 584 COVID-CXRs and 1232 NORMAL-CXRs. We combined the dataset from different resources as shown in Table 1. Then, we manually removed the images with the lateral view of the CXRs and also excluded outliers with distortion and duplicate images.

## 4. Results

**Figure 8:**
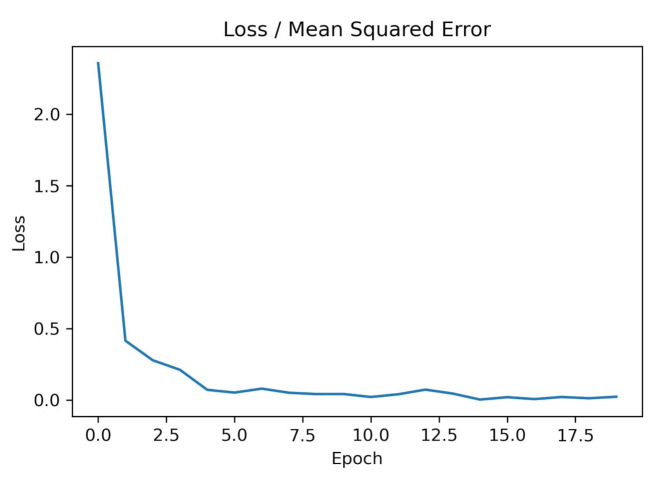
The loss curve for a single CNN model (VGG16) for 20 epochs.

In this section, we outline our results for the detection of COVID-19 CXR images. The results were evaluated with reference to the classification accuracy of the CNN models. The total number of samples was 760 (280 COVID-CXR and 480 Normal-CXR). We analyzed our results using two different types of datasets: (1) using completely original images, and (2) using 50% original images and 50% synthetic images. Using (1), an accuracy of 99.38% was achieved, and with (2), an accuracy of 99.54% was achieved. The results are summarized in Table 2. Each CNN model was trained over 20 epochs. The number of epochs was chosen as 20 because the loss converges after roughly 20 epochs. Figure 8 shows one such loss graph to highlight the point. This training process was repeated over 20 iterations and finally the average and standard deviation values were reported for the considered metrics.

The GAN and the different CNN architectures [21,22,23,24] were trained on a GTX 1650 GPU and were carried out using the keras deep learning library. Table 2 shows the classification accuracy of the various CNN models applied to the said two kinds of datasets. It also presents the results of using feature selection on the VGG16 features in the mixed dataset.

In addition, Table 3 presents a comparative assessment of using various state-of-the-art meta-heuristic feature selection algorithms on the VGG16 features. Apart from the HS algorithm, we also use the following algorithms: Particle Swarm Optimization (PSO) [56], Genetic Algorithm (GA), Mayfly optimization Algorithm (MA) [57], Equilibrium Optimizer (EO) [58], Grey Wolf Optimizer (GWO) [59], Gravitational Search Algorithm (GSA) [60] and Sine Cosine Algorithm (SCA) [61]. Finally, we show that the HS algorithm performed the best in the present scenario.

**Figure 9:**
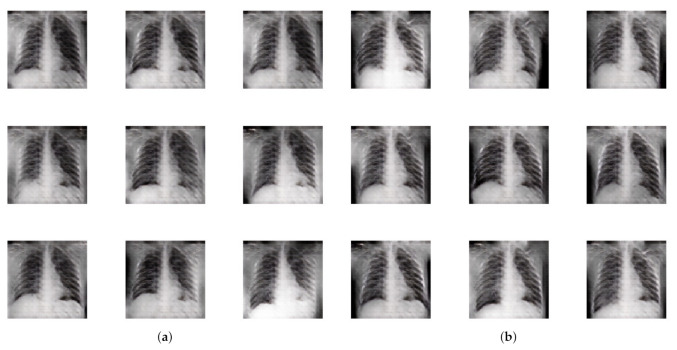
Some synthetic images that were generated using the present approach. (**a**) Synthetic images belonging to the COVID-19 infected CXR class generated by the GAN; (**b**) synthetic images belonging to the normal CXR class generated by the GAN.

Table 4 contains the inception score [62] and the Fretchet inception distance [63] for the images that were generated by the GAN. The Inception Score measures the quality of the images generated by the GAN. It checks how well a pretrained InceptionV3 model can classify the images into one of 1000 known classes. It utilizes the class and marginal probabilities to measure the quality and diversity of the images. However, it does not measure any performance with respect to the real images. For this reason, the Fretchet Inception distance was proposed to tackle the previous issue. It also uses an InceptionV3 model, but measures some statistical properties of the real and synthetic distributions. Figure 9 also presents some synthetic images generated by the present approach. We generated both types of synthetic images, i.e., COVID-CXR and Normal-CXR. The synthetic images generated are shown in Figure 9. We also created a dataset consisting of 500 synthetic COVID-CXR images, which helps in further research endeavors. The data can be found in: https://github.com/yashkarbhari/Generating-COVID-CXR-using-ACGAN (accessed on 27 March 2021).

Finally, in addition to the above two-class classification problem, we also considered a three-class classification problem. This was done to show that the classification approach used in the above two-class task is a general procedure and can be applied to other data as well. Here, the input image needed to be classified as normal, pneumonia-affected or COVID-affected. In this case too, it was observed that the CNN models achieved good accuracy scores. Finally, using HS on the feature obtained by the best CNN model further increased the accuracy score. The results are summarized in Table 5.

## 5. Discussion

It can be observed from Table 2 that the accuracy and AUC either improved or remained the same when including the synthetic data in all of the models. Although the performance of the VGG16 and VGG19 models was similar, we used the features from the VGG16 model. This is because it has fewer parameters, and given the size of the dataset, it is preferable to have fewer parameters to prevent overfitting.

When we applied the more recent and heavyweight models, a noticeable improvement was observed in the performance with the addition of the synthetic data. In the ResNet50 model, we observed an increase of 0.19% in the accuracy. The Xception and Inception models showed greater degrees of improvement: the accuracies improved to 96.91% and 99.17% from 95.41% and 97.37%, respectively. However, even with this improvement, it can be observed that these models were inferior when compared to the VGG16 and VGG19 models.

Since the VGG16 model produced the best results along with VGG19, it was used for the purpose of feature extraction as it had the lesser number of parameters between the two models. The model was trained as usual, but instead of taking the classification probabilities as output, the output of the the second-last dense layer was taken as the features. These features were then used for feature selection.

From Table 3, we can recognize that the HS algorithm managed to reduce the number of features by 62.5% while retaining the accuracy of the VGG16 model. The accuracy also improved to 100.00% from 99.71%. This indicates that there were some irrelevant or correlated features present in the extracted feature vectors, which decreased the classification accuracy of the standalone VGG16 model. It is noted that GSA also achieved an accuracy of 100.00% but selected 65 features in total, which is the highest among all of the feature selection algorithms considered. Furthermore, SCA obtained the maximum reduction value of 70.3% but had a low accuracy of 92.10%. Furthermore, we note that all of the algorithms had a 100% accuracy score on the validation set, whereas the performances on the test set varied.

Figure 9 shows some synthetic images for a qualitative assessment of the output. There was a significant difference between the features of different images, indicating that the GAN provides varied outputs. Furthermore, the inception score mentioned in Table 4 establishes that images in two different classes are distinguishable from each other. The Fretchet distance, however, seems to be on the higher side, indicating that some improvement may be possible. This can be explored in future work.

Finally, Table 5 shows the results of the the present detection approach on a three-class classification task with normal, pneumonia affected and COVID-19 affected CXRs. In this case, the accuracy of the CNN models dropped as expected due to the increase in the number of classes. However, it is noted that a feature selection stage still managed to improve the model performance. The detection pipeline is a general framework and there are no strict restrictions. Hence, one could apply the same process to, for example, CT scan images [64], and even other areas such as those discussed in [65].

One limitation of the present approach is that it may not be able to detect COVID-19 from images of the patients who are in the early stages of infection. This is mainly due to the fact that there may be minor or no significant artifacts present in the CXRs of an individual in the early stages of infection. As a result, this and other similar CNN based approaches will fail to discriminate such cases. Another limitation that is relevant for GAN based synthetic data generation is the quality of the dataset used as a reference for the GAN. The quality and variety of the synthetic images depend directly on the input images that are supplied to the GAN. If the input images do not reflect the proper data distribution, then the GAN will output unsatisfactory images that may be highly similar to each other. In general, GANs require quite a large quantity of data to be able to produce the desired results. Another issue with GANs is their relative instability while training, which may result in mode collapse even with the proper data.

## 6. Conclusions

The rapid spread of the coronavirus has badly affected the healthcare systems around the world. Hence, along with medical practitioners, computer scientists are also trying hard to come up with alternative solutions. In doing so, medical images such as CXRs are being used to identify COVID-19 patients. However, the main limitation of the DL based methods used to serve this purpose is the non-availability of an extensive dataset to train the models. To this end, in this paper, we presented an ACGAN based approach for generating synthetic COVID-19 afflicted CXRs. Additionally, the generated images were used to train some state-of-the-art CNNs to detect COVID-19 in the input X-rays. This was done to evaluate the quality of the generated images. The results illustrate that the CNNs can consistently achieve over 98% classification accuracy over 20 iterations which confirms the high quality of the images and the robustness of the method. We also used the HS algorithm for feature selection on the features extracted by the VGG16 model. We achieved an accuracy of 100% while reducing the number of features by more than 60%. This shows that the HS algorithm helps in removing redundant and correlated features.

An obvious limitation of the present work is the comparatively small dataset that was used for training the GAN. Larger and more varied datasets can be utilized to improve the diversity and the quality of the generated synthetic images. Furthermore, the ACGAN architecture was chosen based on both time and resource constraints. Therefore, variation in the architectures of the generators and discriminators can also be explored to obtain better results. In addition to this, various other datasets can be explored as well. As mentioned before, CXRs and other radiological modalities lack significant artifacts in the early stages of infection. Therefore, other relevant physiological data can also be leveraged for better predictions. In particular, the effect of combining datasets is a viable option for exploration. Finally, better variants and modifications of CNNs (such as ensembling, pruning, etc.) can also be explored to obtain better and robust classification performance. The above limitations and research directions can therefore be handled in future work.

## Figures and Tables

**Table 1:** A summary of the datasets that were used in this work.

Dataset	COVID-19	Normal
covid-chestxray-dataset [55]	1147	0
COVID-chestxray-dataset Initiative ^a^	55	0
Actualmed COVID-19 Chest X-ray Dataset Initiative ^b^	247	0
COVID-19 Radiography Database ^c^	1200	1341

^a^ https://github.com/ieee8023/covid-chestxray-dataset; ^b^ https://github.com/agchung/Actualmed-COVIDchestxray-dataset; ^c^ https://www.kaggle.com/tawsifurrahman/covid19-radiography-database; all URLs accessed on 27 March 2021.

**Table 2:** A summary of the results obtained by the classifiers considered in this work. The results were reported after performing 20 iterations. Values in the table are in the format of mean ± std. dev.

Dataset	Model	Accuracy (%)	Precision (%)	Recall (%)	F1-Score (%)	AUC (%)
Original Data + Synthetic Data	VGG16	99.54 ± 0.40	99.54 ± 0.40	99.54 ± 0.40	99.54 ± 0.40	99.92 ± 0.17
	VGG19	99.53 ± 0.34	99.53 ± 0.34	99.53 ± 0.34	99.53 ± 0.34	99.94 ± 0.08
	ResNet50	99.48 ± 0.43	99.48 ± 0.43	99.48 ± 0.43	99.48 ± 0.43	99.85 ± 0.19
	Xception	96.91 ± 1.04	96.91 ± 1.04	96.91 ± 1.04	96.91 ± 1.04	99.48 ± 0.26
	InceptionV3	99.17 ± 0.39	99.17 ± 0.39	99.17 ± 0.39	99.17 ± 0.39	99.80 ± 0.21
	VGG16 + HS	100.00 ± 0.00	100.00 ± 0.00	100.00 ± 0.00	100.00 ± 0.00	100.00 ± 0.00
Original Data	VGG16	99.36 ± 0.29	99.36 ± 0.29	99.36 ± 0.29	99.36 ± 0.29	99.65 ± 0.48
	VGG19	99.38 ± 0.37	99.38 ± 0.37	99.38 ± 0.37	99.38 ± 0.37	99.82 ± 0.35
	ResNet50	99.29 ± 0.52	99.29 ± 0.52	99.29 ± 0.52	99.29 ± 0.52	99.71 ± 0.31
	Xception	95.41 ± 1.08	95.41 ± 1.08	95.41 ± 1.08	95.41 ± 1.08	99.10 ± 0.80
	InceptionV3	97.37 ± 1.07	97.37 ± 1.07	97.37 ± 1.07	97.37 ± 1.07	99.13 ± 0.46

**Table 3:** A comparison of the performances of various state-of-the-art feature selection algorithms on the mixed dataset (50% original data and 50% synthetic data). The VGG16 features of initial dimension 128 were used.

Feature Selection Algorithm	Accuracy (%)	No. of Features	Reduction (%)
PSO [56]	97.36	43	66.4
GA	97.36	46	64.1
MA [57]	97.36	62	51.6
EO [58]	97.36	51	60.2
GWO [59]	97.36	46	64.1
GSA [60]	100.00	65	49.2
SCA [61]	92.10	38	70.3
HS [25]	100.00	48	62.5

**Table 4:** The inception score and the Fretchet inception distance for the images that were generated by the GAN.

Inception Score	Fretchet Inception Distance
2.508 ± 0.125	50.67 ± 8.127

**Table 5:** A summary of the results obtained by the classifiers on the three-class classification task. The input image was to be classified as normal, pneumonia-affected or COVID-affected.

Model	Accuracy (%)	F1 Score (%)	AUC (%)
VGG16	94.77	93.20	99.39
VGG19	93.91	92.70	99.29
ResNet50	96.97	96.88	99.68
Xception	91.08	92.41	99.27
InceptionV3	92.64	92.85	99.56
VGG16 + HS	100.00	100.00	100.00

## Data Availability

Data available in public repositories were used in this study for training the models. The synthetic data generated can be found at: https://github.com/yashkarbhari/Generating-COVID-CXR-using-ACGAN (accessed on 27 March 2021).
